# Physics-Guided Multi-GSO Spectral Filtering for Degradation-Aware Automotive Radar Point-Cloud Detection

**DOI:** 10.3390/s26154714

**Published:** 2026-07-24

**Authors:** Xiuping Li, Xiyan Sun, Yuanfa Ji, Jingjing Li, Wentao Fu, Songke Zhao, Wenbin Liang, Xizi Jia, Jian Liu

**Affiliations:** 1 School of Information and Communication, Guilin University of Electronic Technology, Guilin 541004, China; lxp863@gmail.com (X.L.); jiyuanfa@guet.edu.cn (Y.J.); lijingjing@guet.edu.cn (J.L.); fuwentao@guet.edu.cn (W.F.); zhaosongke@guet.edu.cn (S.Z.); liangwenbin@guat.edu.cn (W.L.); xizijia@guet.edu.cn (X.J.); 2 Guangxi Key Laboratory of Precision Navigation Technology and Application, Guilin University of Electronic Technology, Guilin 541004, China; liujian2024@hit.edu.cn; 3 School of Aerospace, Harbin Institute of Technology, Shenzhen 518055, China

**Keywords:** automotive radar, autonomous driving, radar point cloud, object detection, sensor degradation, intelligent transportation systems, graph signal processing, graph neural network, spectral filtering, Doppler velocity

## Abstract

Automotive millimeter-wave radar produces sparse point clouds with Doppler velocity and radar cross-section (RCS), but graph detectors typically use a shared representation for semantic prediction and box regression despite their different propagation requirements. We propose multi-GSO spectral filtering (MGSF), a residual module that filters radar features over geometry-, Doppler-, and RCS-defined graph shift operators and fuses diffusion and residual components with a node-adaptive gate. MGSF-TD applies full multi-GSO refinement to semantic prediction and geometry-only refinement to box regression. On the complete RadarScenes validation set, MGSF-TD improves the official RadarGNN checkpoint from 60.19 to 60.59 mAP and from 74.06 to 75.10 mean foreground F1 (FG-F1). Across three MGSF-TD training seeds, the FG-F1 margin under RCS noise increases from +1.15 at 3 dBsm to +2.38 at 20 dBsm; seed-42 full-validation mAP margins are +0.24, +1.07, and +1.82. Controls show that geometry-only diffusion explains part of the gain and the RCS operator contributes most clearly at low-to-moderate noise, whereas a parameter-matched widened baseline matches or exceeds MGSF-TD under severe RCS and Doppler corruption. Cross-sensor diagnostics reproduce the Doppler failure trend but not the severity-dependent RCS gain. MGSF-TD therefore offers a balanced, physically interpretable operating point rather than a universal robustness gain.

## 1. Introduction

Millimeter-wave (MMW) radar is a core sensor for intelligent vehicles. It works in darkness and adverse weather, and it directly measures the Doppler velocity of each return [1]; large-scale annotated point-cloud datasets such as RadarScenes have made this modality accessible to learning-based perception [2]. Radar therefore complements cameras and LiDAR in all-weather perception. Its point clouds are, however, difficult to exploit directly. They are much sparser than LiDAR scans, contain multipath and specular clutter, and include missed returns associated with radar cross-section (RCS) variation.

Radar’s measurement richness is inseparable from its measurement uncertainty. Each return carries physical measurements, including position, RCS, Doppler velocity, and time. These measurements are not interchangeable with geometric coordinates. Their errors come from the sensor process itself: RCS varies with material and aspect angle, localization is limited by range and angular resolution, Doppler can be affected by compensation or reconstruction, and weak returns can disappear. We therefore study radar point-cloud detection under clean inputs and under physically motivated sensor degradation.

Graph neural networks (GNNs) naturally represent sparse and irregular radar point sets [3,4]. RadarGNN [3] constructs a *k*-nearest-neighbor (*k*-NN) graph and jointly predicts point semantics and bird’s-eye-view (BEV) boxes from one shared representation, a design typical of point-cloud graph detectors [3,5]. Standard message passing, however, does not explicitly control the propagation components of graph signals. In a radar graph, diffusion components encode coherent object structure, whereas residual components mark boundaries, clutter, and velocity discontinuities. Uncontrolled aggregation can therefore suppress localization cues or propagate clutter into a target representation.

Graph signal processing (GSP) offers tools for describing these propagation effects [6,7]. Polynomial graph filters, including Chebyshev [8] and Bernstein [9] formulations, avoid eigendecomposition and scale to large graphs. Existing point-cloud filters usually operate on one geometric graph and apply the same component mixing to every node. This design overlooks the multi-attribute structure of radar, in which geometry, Doppler velocity, and RCS provide distinct physical relations between returns.

Detection also imposes different requirements on semantic prediction and box regression. Semantic prediction can benefit from Doppler- and RCS-consistent aggregation, whereas box regression depends strongly on preserved geometric boundaries. A shared filtered representation may therefore improve classification while weakening localization. This task-component conflict motivates separate spectral paths for the two prediction heads.

We address these limitations with multi-GSO spectral filtering (MGSF), which constructs graph shift operators (GSOs) from geometry, Doppler velocity, and RCS. The module separates diffusion and residual components, projects each component, and performs node-adaptive fusion. Its task-decoupled form, MGSF-TD, applies full multi-GSO refinement to semantic prediction and geometry-focused refinement to box regression. We evaluate clean detection, controlled sensor degradation, and the limits of the proposed operator structure. The reliability of perception under sensor faults is an active concern [10]; our earlier work examined bounded graph conditioning for degraded LiDAR detection [11]. The scope of this paper should be stated precisely. It is a module-level methodological contribution to the radar perception layer of autonomous-driving and intelligent transportation systems: it studies how radar-physical measurements should shape graph propagation inside a point-cloud detector, and how the resulting detector behaves when those measurements degrade. It does not cover full driving pipelines, multi-sensor fusion, or downstream vehicle decision-making; Section 6 positions the module within these larger deployment questions.

The main contributions are as follows.

1. **A multi-GSO spectral filtering module for automotive radar sensing.** We introduce a residual filter with geometry-, Doppler-, and RCS-defined GSOs. Component-specific projections and a node-adaptive gate fuse complementary diffusion–residual components, while identity initialization preserves the pre-trained detector at the start of fine-tuning.
2. **Task-decoupled spectral detection with a balanced clean-data trade-off.** We identify a conflict between Doppler-sensitive semantic aggregation and geometry-sensitive localization. MGSF-TD separates the two spectral paths and improves both clean mAP (60.19 to 60.59) and FG-F1 (74.06 to 75.10) under the official RadarGNN pipeline.
3. **An operator-level degradation diagnostic with explicit boundaries.** We combine single-attribute and sensor-structured corruptions with augmentation, GSO-subset, and parameter-budget controls. The analysis separates generic diffusion smoothing from RCS-defined neighborhood effects and identifies the regimes where capacity or Doppler corruption dominates. Cross-sensor diagnostics then test which trends persist under different radar sensors and dataset-native RCS behavior.

## 2. Related Work

**Radar point-cloud object detection.** Deep learning for automotive radar perception has grown into a substantial literature, reviewed in [12,13,14]. Early systems operate on range-Doppler images or occupancy grids [15,16]. Methods that process sparse detections directly include PointNet-style encoders [17] and pillar encoders adapted from LiDAR, a line that continues to evolve toward lightweight transformer-augmented pillar detectors [18]. High-resolution 4D radar has spurred dedicated detectors and datasets [19,20,21,22] and raw-radar multi-task learning [23,24]. These families differ in where they enter the radar signal chain. Raw-data detectors such as TransRAD [25] operate on range–azimuth–Doppler tensors before threshold-based point extraction, which preserves weak returns but ties the model to one sensor’s data format and resolution. Point-cloud detectors, including RadarGNN and MGSF, instead consume the sparse detection lists that production automotive radars output; this inherits the information loss of on-chip thresholding but applies across sensors that share the point-cloud interface, a property our cross-sensor diagnostics in Section 4.8 rely on directly. Among graph methods, RadarGNN [3] reaches strong RadarScenes accuracy with spatial *k*-NN message passing and a dual semantic/regression head, and graph models continue to be explored for radar point clouds, including for target classification [26]. Because implementation and post-processing details differ across papers, published accuracy values are not directly comparable; our primary comparison therefore re-evaluates the public RadarGNN checkpoint with the same preprocessing, rotated NMS, and metrics as MGSF. RPFA-Net [27] adds self-attention pillar aggregation, and RCBEVDet [28] fuses camera and radar in BEV space, as do attention-based camera–radar fusion networks for adverse weather [29]; radar–camera fusion has also been extended beyond detection to multi-object tracking with explicit robustness to single-sensor failure [30], and such fusion systems are natural consumers of the per-point radar refinement studied here. Camera–LiDAR fusion detectors for complex traffic scenes [31], multi-task fusion frameworks that couple 3D detection with occupancy perception [32], and camera-based multi-object perception systems [33] illustrate the breadth of the surrounding autonomous-driving perception literature into which a radar-side module must eventually integrate. Dynamic graph methods such as DGCNN [34] recompute the neighborhood graph in feature space at each layer rather than fixing it. Attribute-defined spectral operators, and their behavior under controlled radar degradation, remain unexamined in these approaches; those two questions define the scope of this work.

**Radar robustness and adverse sensing conditions.** Robustness of 3D perception to input corruptions has been benchmarked primarily for LiDAR and cameras [35], while adverse-weather sensing has motivated multimodal datasets and radar-assisted perception systems [22,29,36]. Radar point clouds add modality-specific measurement effects, including RCS fluctuation, Doppler uncertainty, angular-resolution-limited localization, and sparse missed returns. Existing studies generally evaluate end-task degradation or sensor fusion; controlled, operator-level analysis of how these physical attributes define radar graph propagation remains limited.

**GNNs for point clouds.** PointNet++ [37] and DGCNN [34] show that multi-scale local features improve 3-D understanding, and Point-GNN [5] is a graph detector for LiDAR. These methods emphasize spatial message passing. Their graphs are driven by proximity and lack explicit component control, so the over-smoothing of boundary cues that matters for radar regression is not addressed.

**Graph signal processing and spectral filters.** Spectral graph convolution [38] became scalable with ChebNet [8]. GCN [39] uses a first-order approximation, GPR-GNN [40] learns polynomial weights for heterophily, and BernNet [9] uses a non-negative Bernstein basis; recent work analyzes the expressive power of spectral GNNs [41] and couples them with transformers [42]. These filters are shared across all nodes and operate on a single graph. We differ in two ways. We apply several physics-defined GSOs at once, and we make the component mixing adaptive per node. Multi-view graph learning [43] and physics-informed GNNs [44] combine several adjacencies or domain constraints, but they neither exploit the Doppler and RCS physics specific to radar nor analyze the resulting robustness structure, which is the focus here. Table 1 summarizes how MGSF differs from representative graph spectral filtering, multi-graph, and graph detection methods in graph construction, filtering strategy, and robustness objective.

**Radar point-cloud sensor data processing.** Point-cloud processing has an extensive literature on denoising, filtering, semantic segmentation, object detection, and geometric reconstruction, with continuing advances in, for example, ground extraction for unstructured environments [45] and multi-scale point-cloud registration [46]. Most of it was developed for LiDAR or photogrammetric point clouds, where geometry is the dominant cue, and LiDAR-oriented deep models now also serve as feature extractors in adjacent perception subsystems such as online LiDAR–camera calibration [47]. Automotive radar point clouds are different sensor data. The differences are worth stating explicitly. Radar point clouds are far sparser than LiDAR sweeps; each return carries Doppler velocity and RCS as intrinsic physical measurements that LiDAR does not provide; multipath and specular reflections create clutter returns that are plausible detections of non-existent structure rather than statistical outliers; and the comparatively coarse angular resolution of automotive radar makes positional uncertainty anisotropic and range-dependent. LiDAR-derived detection architectures therefore transfer only partially, and radar pipelines often add a sensing-side enhancement stage; for example, point-cloud enhancement models can densify sparse mmWave returns before downstream inference [48]. MGSF targets this radar-sensor setting by building graph shift operators directly from these measurements and by studying how the resulting filter behaves when each measurement is degraded.

## 3. Materials and Methods

### 3.1. Dataset and Evaluation Protocol

**Dataset.** RadarScenes [2] is a large-scale automotive radar dataset recorded with four vehicle-mounted sensors. It contains 158 sequences with point-level semantic labels and track-level object annotations. We follow the official RadarGNN preprocessing: 500 ms temporal accumulation, a 200×100 m bird’s-eye-view (BEV) crop, *k*-NN graph construction, and directed edges with relative position as edge features. This yields 20,449 training and 5003 validation graphs. The *k*-NN graph uses k=20, following RadarGNN.

**Metrics.** We report mean average precision (mAP) at point-IoU ≥0.3 over the five foreground classes (car, pedestrian, pedestrian group, two-wheeler, large vehicle), and mean foreground F1 (FG-F1), the unweighted mean of per-class F1 over the same five classes. BEV box post-processing uses exact rotated non-maximum suppression (implemented by detectron2.layers.nms_rotated, detectron2 version 0.6.). The public RadarGNN model_02 checkpoint yields 60.19 mAP under this protocol and serves as the controlled baseline throughout. Methods that use different sensor configurations or datasets are not directly comparable, so we do not tabulate them as baselines. For example, RPFA-Net [27] targets 4D-radar pillar input and cannot consume the RadarScenes 2D detection lists without re-engineering its input stage.

**Training.** All MGSF variants are fine-tuned from the RadarGNN model_02 checkpoint (translation-invariant setting) with Adam, batch size 5, weight decay $5\times 10^{-6}$, Huber box loss weight 0.5, and an exponential learning-rate decay of 0.98 per epoch. Stage 1 trains the full three-GSO backbone for at most 10 epochs at learning rate $10^{-4}$. It stops early if the validation loss does not improve for three epochs. Stage 2 loads the best Stage 1 checkpoint, freezes the backbone MGSF blocks, and trains only the task-decoupled heads (11k parameters) for at most 20 epochs at learning rate $10^{-4}$ with patience 5. The saved checkpoint with the lowest validation loss is used for evaluation. MGSF uses diffusion order K=2 and bandwidths $\sigma_{\mathrm{geo}}\;=\;4.0$ m, $\sigma_{\mathrm{vel}}\;=\;1.0$ m/s, and $\sigma_{\mathrm{rcs}}\;=\;10.0$ dBsm; the velocity and RCS bandwidths are dataset-calibrated choices whose rationale and sensitivity are given in Section 3.5 and Appendix Table A5. Unless otherwise specified, clean detection, ablations, per-class analysis, full-validation RCS mAP, and the WideCap control use the pre-declared training seed 42. The central FG-F1 robustness benchmark reports MGSF-TD over seeds 42, 7, and 123. The statistical scope is detailed in Section 3.7. Corruption samples use evaluation RNG seed 0 and are shared between the compared models.

### 3.2. Radar Graphs and Spectral Preliminaries

A radar frame of *N* points gives each point *i* raw attributes: BEV position $p_{i}\in R^{2}$, Doppler velocity $v_{i}\in R^{2}$, RCS $r_{i}\in R$, and time index $\tau_{i}$. The RadarGNN baseline already takes RCS and velocity as node features, so the comparison against it isolates the contribution of the multi-GSO spectral filtering rather than that of adding multi-attribute inputs. A directed *k*-NN graph G=(V,E) connects each point to its *k* spatially closest neighbors, following RadarGNN. At GNN layer *l*, let $H^{\left(l\right)}\in R^{N\times d}$ be the node embedding at layer *l*. MGSF treats $H^{\left(l\right)}$ as a graph signal and applies a residual correction built from graph diffusion components before layer l+1; Remark 1 states the precise, limited spectral sense in which this correction acts as a filter.

Because RadarGNN uses directed *k*-NN edges, the symmetric normalized Laplacian spectrum is not directly defined on the propagation graph. MGSF therefore implements its smoothing step as a degree-normalized directed diffusion operator $S:H_{i}\mapsto \left(\sum_{j}w_{ji}H_{j}\right)/\left(\sum_{j}w_{ji}\right)$, and uses $S^{K}H$ as a *K*-step diffusion component. Its complementary residual, $H-S^{K}H$, captures information not reconstructed by that diffusion. Where a spectral diagnostic requires eigenvalues we form the symmetrized adjacency $A_{\mathrm{sym}}=\left(A+A^{⊤}\right)/2$ and its normalized Laplacian $L=I-D^{-1/2}A_{\mathrm{sym}}D^{-1/2}$ ($D_{ii}=\sum_{j}{\left(A_{\mathrm{sym}}\right)}_{ij}$), whose eigenvalues lie in [0,2]. This diagnostic graph is used only to interpret the operator response; the implemented MGSF block uses directed diffusion and does not assume a global eigendecomposition. The per-component learnable transforms are linear projections, not learned filter coefficients.

**Remark** **1** (Diffusion–residual decomposition)**.** *Because the directed k-NN diffusion operator is generally non-symmetric, MGSF does not assume a global eigendecomposition. We interpret $S_{m}^{K}H$ as a K-step diffusion component and $H-S_{m}^{K}H$ as its residual component. On the symmetrized diagnostic graph, their responses are $h_{\mathrm{diff}}\left(\lambda \right)={\left(1-\lambda \right)}^{K}$ and $h_{\mathrm{res}}\left(\lambda \right)=1-{\left(1-\lambda \right)}^{K}$. These are operational graph-filter diagnostics, not ideal monotone low- and high-pass filters over the full normalized Laplacian interval [0,2], especially when the symmetrized adjacency has negative eigenvalues. Accordingly, any use of “low” or “high” is only a shorthand for diffusion-dominant and residual-dominant components.*

### 3.3. Multi-GSO Spectral Filtering: What the Module Is

**Figure 1:**
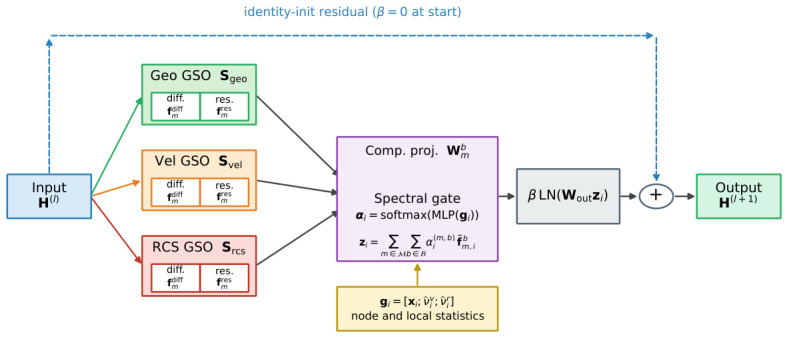
Overview of the MGSF block inserted after each RadarGNN convolutional layer. Three GSOs (Geo, Vel, RCS) produce diffusion and residual components. Component-specific projections and a per-node gate fuse them into a residual correction. Here *m*, *b*, and *i* index the GSO, component, and graph node, respectively.

Figure 1 overviews the MGSF block, which is inserted after each RadarGNN convolution. The block computes three radar-physics GSOs with no learned parameters, extracts a diffusion component and its residual per GSO, projects each component, and fuses all components per node into a residual correction. We describe the three GSOs first (what the module is), then the design rationale (why it works), and defer performance to Section 4.1 and Section 4.2 (how well it works).

We use M={geo,vel,rcs} for the GSO index set and B={diff,res} for the component index set. For m∈M and b∈B, $S_{m}$ denotes a directed diffusion GSO, $f_{m}^{b}$ its component output, and $W_{m}^{b}\in R^{d\times d}$ the corresponding projection. At node *i*, $g_{i}\in R^{7}$ is the gate input, $\alpha_{i}^{\left(m,b\right)}$ is a scalar softmax weight, and $z_{i}\in R^{d}$ is the fused feature. $W_{\mathrm{out}}\in R^{d\times d}$ is the output projection, β∈R is a learnable residual scale, and LN and GELU denote layer normalization and the Gaussian error linear unit, respectively.

The geometric GSO weights edge (j,i) by $w_{ji}^{\mathrm{geo}}=\exp(-∥\Delta p_{ji}{∥}_{2}^{2}/\sigma_{\mathrm{geo}}^{2})$, where $\Delta p_{ji}$ is the relative position. The Doppler velocity GSO weights it by $w_{ji}^{\mathrm{vel}}=\exp(-∥v_{j}-v_{i}{∥}_{2}^{2}/\sigma_{\mathrm{vel}}^{2})$, which links co-moving points that belong to the same rigid object. The RCS-similarity GSO weights it by $w_{ji}^{\mathrm{rcs}}=\exp(-|r_{j}-r_{i}\left|/\sigma_{\mathrm{rcs}}\right)$, using an $L_{1}$ (Laplacian) kernel rather than a squared-distance kernel because RCS contains occasional large specular outliers to which the slower-decaying kernel is more robust.

### 3.4. Design Rationale

The design separates three complementary relations between radar returns. The geometric diffusion component represents spatial coherence, the Doppler diffusion component aggregates co-moving returns, and the RCS diffusion component represents reflectivity coherence. Their residual components emphasize information not reconstructed under each relation. Doppler and RCS differences also alter the relative edge weights before degree normalization, changing which neighbors dominate diffusion. Separate component projections allow these signals to contribute differently during fusion.

Formally, for each GSO *m* the *K*-step diffusion component and its residual are

(1) $$f_{m}^{\mathrm{diff}}=S_{m}^{K}H,\;f_{m}^{\mathrm{res}}=H-f_{m}^{\mathrm{diff}}.$$

At node *i*, each component is transformed as

(2) $${\tilde{f}}_{m,i}^{b}=\mathrm{GELU}\;\left(\mathrm{LN}\;\left(W_{m}^{b}f_{m,i}^{b}\right)\right),$$

with $W_{m}^{b}$ specific to the (m,b) pair.

A lightweight gate fuses the six components at each node. It is a context-dependent fusion mechanism rather than the main source of model capacity. The learned weights remain close to uniform in practice (Section 4.7), leaving most discrimination to the component-specific projections. The gate uses raw radar and graph attributes with local velocity and RCS dispersion. Specifically, let $x_{i}={\left[r_{i},v_{x,i},v_{y,i},\tau_{i},\deg_{i}\right]}^{⊤}\in R^{5}$ contain RCS, Cartesian Doppler velocity, timestamp, and graph degree. Over the incoming neighborhood $N_{i}$, we compute

(3) $$\nu_{i}^{v}=\frac{1}{|N_{i}|}\sum_{j\in N_{i}}{∥v_{j}-v_{i}∥}_{2}^{2},\;\nu_{i}^{r}=\frac{1}{|N_{i}|}\sum_{j\in N_{i}}{\left(r_{j}-r_{i}\right)}^{2}.$$

Their scale-normalized forms are denoted by ${\hat{\nu }}_{i}^{v}$ and ${\hat{\nu }}_{i}^{r}$. The gate input and its six weights are

(4) $$g_{i}=\mathrm{LN}\left(\left[x_{i};{\hat{\nu }}_{i}^{v};{\hat{\nu }}_{i}^{r}\right]\right),\;\alpha_{i}=\mathrm{softmax}\left(\mathrm{MLP}\left(g_{i}\right)\right)\in R^{6}.$$

Here LN is a per-node layer normalization over the seven gate inputs. The softmax normalizes over the six (GSO, component) pairs, so the six gate weights are positive and sum to one at each node. The raw attributes $r_{i}$, $v_{x,i}$, $v_{y,i}$, $\tau_{i}$, and $\deg_{i}$ are the same preprocessed node features used by RadarGNN; the two local dispersion terms are divided by their batch standard deviations before LN. The gate MLP has one hidden layer of size max(d/4,16) with GELU activation and outputs one logit per GSO–component pair. The fused embedding is $z_{i}=\sum_{m\in M}\sum_{b\in B}\alpha_{i}^{\left(m,b\right)}{\tilde{f}}_{m,i}^{b}$.

The block output is $H_{i}^{\prime }=H_{i}+\beta \;\mathrm{LN}\left(W_{\mathrm{out}}z_{i}\right)$, where the scalar β is initialized to 0. The block is thus an exact identity at the start of fine-tuning, which preserves any pre-trained checkpoint and prevents early instability.

### 3.5. Task-Decoupled Refinement (MGSF-TD)

Multi-GSO filtering creates a task-component conflict, stated below as a mechanistic hypothesis derived from the velocity-GSO construction.

**Hypothesis** **1** (Task-component conflict)**.** *The velocity GSO strengthens edges between co-moving, same-class returns and can therefore aid semantic prediction. Now consider spatially adjacent classes $c_{\mathrm{slow}}$ (pedestrians) and $c_{\mathrm{static}}$ (clutter) with velocity separation δv. As the ratio $\sigma_{\mathrm{vel}}/\delta v$ increases, the cross-class edge weight $w_{ji}^{\mathrm{vel}}=\exp\left[-{\left(\delta v/\sigma_{\mathrm{vel}}\right)}^{2}\right]$ approaches one. When $\sigma_{\mathrm{vel}}\gg \delta v$, these cross-class edge weights remain close to unity, and the edges can inject off-class spatial offsets into box regression, thereby blurring the estimated object extent.*

This mechanism is sufficient but not universal. The effect also depends on point density, the relative-velocity distribution, and the cross-class points selected by the spatial *k*-NN graph.

Hypothesis 1 predicts two observable effects. Adding Doppler components should trade FG-F1 against mAP, with the largest AP loss for pedestrians because their velocity is closest to static clutter. We test both predictions in Section 4. The final spectral refinement branches before the prediction heads. The classification head receives full multi-GSO refinement $H_{\mathrm{sem}}=\mathrm{MGSF}\left(H;\left\{S_{\mathrm{geo}},S_{\mathrm{vel}},S_{\mathrm{rcs}}\right\}\right)$, and the regression head receives geometry-only refinement $H_{\mathrm{bbox}}=\mathrm{MGSF}\left(H;\left\{S_{\mathrm{geo}}\right\}\right)$. Both branches share the backbone, only the final refinement is specialized, and the branch adds about 11k parameters. A two-stage protocol stabilizes training: Stage 1 converges the backbone, and Stage 2 freezes it and trains only the task-decoupled heads.

The velocity bandwidth $\sigma_{\mathrm{vel}}$ must match the velocity scale that separates classes. In RadarScenes, ego-motion-compensated pedestrian speeds are 0.5 to 2 m/s while the static clutter lies near 0. At $\sigma_{\mathrm{vel}}\;=\;3$ m/s a pedestrian-to-clutter edge has weight exp(−1/9)≈0.90, almost indistinguishable from an intra-object edge; at $\sigma_{\mathrm{vel}}\;=\;1$ m/s the same edge has weight exp(−1)≈0.37, which separates the classes. We therefore choose $\sigma_{\mathrm{vel}}$ so that the kernel moderately suppresses edges at a representative pedestrian–clutter separation $\delta v_{\mathrm{ref}}$; for RadarScenes, $\delta v_{\mathrm{ref}}\approx 1$ m/s and $\sigma_{\mathrm{vel}}\;=\;1$ m/s give a cross-class weight near 0.37. This is a dataset-calibrated criterion, not a universal closed-form bandwidth rule. We set $\sigma_{\mathrm{rcs}}\;=\;10$ dBsm for the same reason: it avoids a hard RCS threshold while preserving a measurable clean-graph contrast between same-class foreground edges and foreground-to-background edges (Appendix Table A2).

### 3.6. Robustness Evaluation Protocol

**Design note: fixed candidate topology.** Across all corruption evaluations the *k*-NN candidate edge index is never rebuilt from corrupted coordinates: position-type corruptions retain every original candidate edge and enter only through node and edge features, and point dropout removes just the edges incident to dropped nodes. This deliberate choice isolates feature- and operator-level effects from graph-construction effects, and it is essential for interpreting the position-noise results, where both compared models receive the same perturbed geometry over the same candidate edges.

We evaluate fixed trained models under two degradation families. The idealized family adds zero-mean Gaussian noise to one attribute at a time: RCS amplitude, point position, or Doppler velocity. Each severity uses the native measurement unit and evaluation RNG seed 0, shared by all models. This setting isolates the effect of each attribute. For idealized position noise, node coordinates and relative-position edge attributes are perturbed, while the original *k*-NN edge index is retained. The sensor-structured family applies range-dependent polar localization jitter and random point dropout. The latter is a controlled sparsity stress test, not a complete model of range-, RCS-, and signal-to-noise-dependent detection. Table 2 summarizes the physical interpretation of each mode and the rationale for its severity range.

These corruptions are first-order surrogates, not a radar channel model. Real degradations are scene-coupled: multipath produces spatially structured ghost targets rather than independent per-point noise, mutual interference raises the noise floor and alters the detection list itself, and precipitation attenuates returns while also suppressing weak detections. We use attribute-isolated noise deliberately, because it keeps the operator-level attribution interpretable—each corruption enters through a known subset of the GSOs—and we read the severity grids as spanning mild to beyond-nominal stress in each native unit rather than as calibrated physical scenarios. Scene-coupled effects such as ghost targets and interference are outside the present evaluation and are revisited as a limitation in Section 5.

For range-dependent polar jitter, a point with polar coordinates $\left(\rho_{i},\theta_{i}\right)$ is perturbed as

(5) $$\rho_{i}^{\prime }=\rho_{i}+\epsilon_{\rho ,i},\;\theta_{i}^{\prime }=\theta_{i}+\epsilon_{\theta ,i},\;\epsilon_{\rho ,i}∼N\left(0,\sigma^{2}\right),\;\epsilon_{\theta ,i}∼N\;\left(0,{\left(\frac{\sigma }{\max(\rho_{\mathrm{med}},1)}\right)}^{2}\right),$$

where $\rho_{\mathrm{med}}$ is the median range of the graph. This gives range noise with standard deviation σ and cross-range angular noise scaled to the graph’s typical range. Edge attributes are recomputed from the perturbed coordinates, but the original edge index is retained. For point dropout, a fraction *p* of nodes is removed uniformly at random, all incident edges are pruned and remapped, and at least two nodes are retained for numerical safety; no new *k*-NN graph is built. We report FG-F1 for all corruption modes. Detection mAP is also evaluated on the complete 5003-graph validation split for RCS corruption, where the semantic robustness effect is strongest, with the robustness gap $\Delta_{\mathrm{rob}}=m_{M}GSF-m_{\mathrm{base}}$ for each reported metric *m*. As a mitigation study we also retrain MGSF with light velocity augmentation (zero-mean Gaussian, σ=0.3 m/s, probability 0.5).

### 3.7. Statistical Scope and Uncertainty Reporting

This subsection states explicitly which uncertainty each analysis does and does not quantify. The public RadarGNN release provides one official checkpoint. The clean detection results, ablations, and per-class analysis use a single pre-declared training seed (42); the clean mAP and FG-F1 offsets are therefore descriptive fixed-checkpoint results, not estimates of training-seed significance. For the central FG-F1 robustness benchmark, we additionally report MGSF-TD over three training seeds (42, 7, 123) against the same official RadarGNN checkpoint. This tests whether the MGSF-TD side of the robustness trend is seed-stable, but it is not a paired three-seed baseline comparison. To probe the baseline side, we additionally retrain the plain RadarGNN baseline from scratch with two further seeds under our evaluation environment and repeat the robustness grid (Appendix Table A6). One retrained seed closely reproduces the official checkpoint’s degradation profile at somewhat lower clean accuracy, whereas the other stops at an earlier validation optimum and trades about five clean FG-F1 points for flatter degradation, most visibly under Doppler noise. Neither reaches the official checkpoint’s clean accuracy, so the released checkpoint remains the strongest clean reference and, because MGSF-TD is fine-tuned from it, the paired control. Under the strongest corruptions the flatter seed-7 model can exceed the official checkpoint, so the retrained seeds bracket rather than reproduce its degradation profile; severity-dependent margins are therefore claimed only for the paired official-checkpoint comparison, and baseline training variance is treated as a separate uncertainty source rather than folded into the reported margins. The seed-42 model is used separately for the graph-level bootstrap over the 5003 validation graphs and qualitative figures. That bootstrap quantifies evaluation-set uncertainty for a fixed model pair, not training-seed uncertainty. A widened RadarGNN trained with seed 42 provides the parameter-budget control: it has 2.110M parameters versus 2.091M for MGSF-TD (+0.9%) and uses the same data, features, losses, and training schedule.

## 4. Results

### 4.1. Main Detection Results

Table 3 compares three configurations under exact rotated NMS. MGSF Geo increased mAP by 0.50 and FG-F1 by 0.52. Full three-GSO filtering produced a larger FG-F1 gain (+1.03) but reduced mAP by 0.33. MGSF-TD avoided this trade-off, increasing mAP by 0.40 and FG-F1 by 1.04. MGSF Geo therefore gives the highest clean mAP, whereas MGSF-TD provides the only joint improvement over RadarGNN in both metrics.

### 4.2. Robustness Under Sensor Degradation

FG-F1 is evaluated under single-attribute corruption. MGSF-TD is averaged over three training seeds, whereas RadarGNN uses the official checkpoint.

The RCS margin increases monotonically when MGSF-TD is averaged over its three training seeds, from +1.15 at $\sigma_{\mathrm{rcs}}\;=\;3$ to +2.38 at $\sigma_{\mathrm{rcs}}\;=\;20$ dBsm. The seed-42 model pair, used only for fixed-checkpoint diagnostics, has a larger highest-severity margin of +2.76. A 2000-resample graph-level bootstrap over the 5003 validation graphs gives a 95% confidence interval of [+2.40,+3.10] for that seed-42 difference. We therefore treat the RCS trend as seed-stable on the MGSF side and graph-stable for the fixed seed-42 pair; the from-scratch baseline seed control in Appendix Table A6 probes the baseline side directly, and because the retrained seeds bracket the official degradation profile, the severity trend is claimed only for this paired fixed-checkpoint comparison. RCS variation is an operationally relevant radar degradation [49].

Positional corruption produces no monotonic margin. The absolute gap remains at most 0.62 points, within the MGSF-TD seed standard deviation at every severity. Both models therefore degrade similarly because they receive the same perturbed coordinates and relative-position edge attributes, while the original candidate edge index is retained.

Table 4 summarizes the corresponding corruption results.

Doppler corruption shows the opposite trend. The margin decreases from +1.83 at 0.5 m/s to +0.24 at 1 m/s and −0.63 at 2 m/s. The two larger perturbations are diagnostic stress tests rather than nominal Doppler errors, because radar estimates radial velocity precisely in the frequency domain [1]. They nevertheless identify a clear boundary of the velocity-GSO design.

Velocity augmentation raised FG-F1 at $\sigma_{\mathrm{vel}}\;=\;2$ m/s from 23.1 to 49.8 for MGSF and to 57.6 for RadarGNN (Appendix Table A1). The recovery was therefore not specific to MGSF. The same augmentation did not reproduce the RCS margin and reduced clean and positional accuracy. These results separate a generic Doppler mitigation from the RCS behavior examined below.

The GSO-subset experiment separated generic diffusion smoothing from the RCS-defined operator (Table 5). We treat Geo-only filtering as a same-pipeline generic diffusion-smoothing control: it already produced a margin that increased from +0.72 to +1.90. Adding the velocity GSO did not improve this trend. Adding the RCS GSO contributed a further +0.56, +1.25, and +1.49 as RCS noise increased. The low- and mid-severity increments are the clearest evidence for RCS-neighborhood structure beyond generic smoothing. At $\sigma_{\mathrm{rcs}}\;=\;20$ dBsm, the same increment should be interpreted more cautiously because the RCS edge weights become close to uniformly small (Appendix Table A2), making extra smoothing and capacity plausible contributors.

The raw edge weights provide a diagnostic of how the RCS-defined neighborhood changes under corruption (Appendix A, Table A2). When clean, the mean RCS-GSO weight $w_{ji}^{\mathrm{rcs}}=\exp(-|r_{j}-r_{i}\left|/\sigma_{\mathrm{rcs}}\right)$ is 0.52 on cross-object (foreground-to-background) edges versus 0.62 on intra-class foreground edges, so the operator discounts some cross-object associations. Under $\sigma_{\mathrm{rcs}}\;=\;20$ dBsm corruption every weight collapses to about 0.25, because the injected noise inflates $|r_{j}-r_{i}|$ and shrinks the exponential. Since the diffusion operator is degree-normalized, this collapse does not imply a proportional reduction in branch amplitude. It does imply that the strongest RCS stress case is no longer a clean test of class-discriminative RCS neighborhoods. The GSO-subset ablation therefore supports an RCS-operator contribution, while the edge-weight and WideCap results bound how strongly that contribution can be attributed at the highest severity.

**Figure 2:**
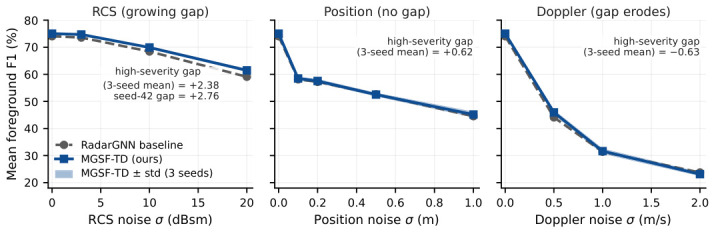
Robustness of MGSF-TD versus the RadarGNN baseline under single-attribute sensor degradation (MGSF-TD: mean over three seeds (42, 7, 123) with ±std band; baseline: official checkpoint). The MGSF-TD margin grows monotonically under RCS-amplitude corruption, shows no severity-amplified trend under common-mode positional corruption, and erodes under strong Doppler corruption, consistent with the operator-structure account in Section 4.2. All three panels share the same vertical scale.

Figure 2 shows the corresponding mean and standard-deviation bands.

Appendix Figure A3 shows the RCS effect on a representative validation scene. Under RCS noise at $\sigma_{\mathrm{rcs}}\;=\;20$ dBsm both methods detect the two objects almost completely when clean (97% and 98% recall), but under RCS noise the baseline foreground recall falls to 67% as it loses the left-hand object to misclassification (red crosses), whereas MGSF-TD holds 94% and keeps that object intact.

### 4.3. Detection mAP and Per-Class Behavior Under RCS Corruption

Whether the RCS robustness extends to box detection, and not only to point semantics, is tested by re-running the complete detection pipeline with exact rotated NMS on all 5003 RadarScenes validation graphs. Models are held fixed and receive identical corrupted inputs. Table 6 reports mAP across RCS severities, and Appendix Table A3 gives the per-class AP at $\sigma_{\mathrm{rcs}}\;=\;20$ dBsm.

MGSF-TD retains a positive detection-mAP margin at every tested RCS severity. The full-validation margin is +0.24 at $\sigma_{\mathrm{rcs}}\;=\;3$ dBsm, +1.07 at 10 dBsm, and +1.82 at the strongest stress setting of 20 dBsm. These results show that the RCS-related effect reaches final box detection rather than remaining confined to point semantics. We interpret the high-severity point together with the capacity control rather than as a standalone proof of RCS-specific superiority. The per-class breakdown localizes the strong-noise improvement: two-wheelers gain +6.20 AP, cars +2.66, and pedestrian groups +1.76; pedestrian AP changes only slightly (+0.20), while large-vehicle AP decreases by 1.75.

### 4.4. Sensor-Structured and Sparsity Degradation

The idealized single-attribute noise isolates each operator, but real radar degradation is structured. Table 7 reports range-dependent localization jitter applied in polar coordinates and a generic random missed-return sparsification test. Only the former is a sensor-structured model; uniform point dropout is retained as a controlled sparsity stress test rather than as a calibrated model of RCS fall-off. Range-dependent jitter behaves like idealized positional corruption: both models receive the same perturbed coordinates and recomputed relative-position edge attributes while their candidate edge indices remain fixed. It therefore degrades both models together with only a small residual gap (+0.67 down to +0.24 as severity rises). Point dropout leaves MGSF-TD a stable positive margin (+1.05 to +1.20 from 10% to 40% dropout), consistent with diffusion components reconstructing each node’s signal from its surviving neighbors. Neither stress test overturns the pattern from the idealized benchmark.

### 4.5. Ablation Study

Each design element contributes in the predicted direction. Table 8 decomposes the model. The velocity GSO alone reduced mAP relative to geometry-only while improving FG-F1, and the full model added FG-F1 but dropped mAP, which is the conflict of Hypothesis 1. Calibrating $\sigma_{\mathrm{vel}}$ to 1.0 m/s lowered the pedestrian-to-clutter edge weight from about 0.90 to about 0.37 and recovered 0.26 mAP. Replacing the gate with uniform weights, or the component-specific projections with shared ones, each reduced mAP, which confirms their role. The uniform gate margin is only 0.12 points, so we read it as directional rather than decisive.

**Bandwidth sensitivity.** The kernel bandwidths are the module’s main hyperparameters, so we quantify how sharply performance depends on them. Appendix Table A5 sweeps the full three-GSO model around the selected operating point (seed 42, one retraining per changed setting): $\sigma_{\mathrm{vel}}$ over 0.5–3.0 m/s and $\sigma_{\mathrm{rcs}}$ over 3–10 dBsm. Clean mAP varies by at most 0.39 points over the whole grid, and every setting except the uncalibrated $\sigma_{\mathrm{vel}}\;=\;3.0$ m/s value analyzed in Section 3.5 and the overly narrow $\sigma_{\mathrm{rcs}}\;=\;3$ dBsm lies within 0.11 points of the selected configuration. The chosen bandwidths therefore sit on a flat plateau, and the physical calibration criterion of Section 3.5 matters mainly for excluding clearly mis-scaled settings rather than for fine-tuning. $\sigma_{\mathrm{geo}}\;=\;4.0$ m was fixed throughout to match the spatial scale of the *k*-NN neighborhoods and was not swept.

### 4.6. Per-Class Analysis

The per-class results localized the main clean-data loss to pedestrians (Appendix Table A4). Full MGSF reduced pedestrian AP by 2.38 points, consistent with the task-component hypothesis. MGSF-TD improved two-wheeler AP by 1.84 points over RadarGNN but left pedestrian AP 2.03 points below the baseline. Section 5 discusses this remaining failure.

### 4.7. Spectral Diagnostics and Complexity

**Figure A1:**
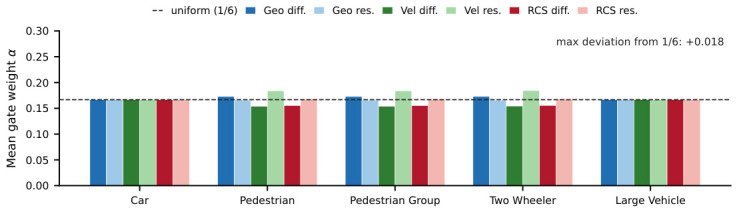
Class-wise mean spectral-gate weights over the six (GSO, component) combinations, extracted from 200 RadarScenes validation graphs. The learned gate stays close to uniform while component-specific projections carry most of the discrimination. The vertical axis spans the full [0,0.3] range; the maximum deviation from the uniform value 1/6 is 0.018.

**Figure A2:**
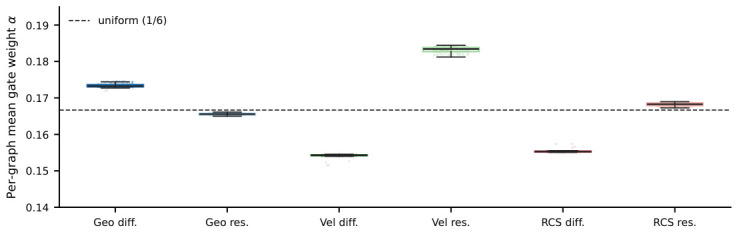
Scene-level stability of the spectral gate. Each box summarizes the per-graph mean gate weight of one (GSO, component) pair over 500 validation graphs sampled with a uniform stride across the complete 5003-graph validation split, so all recording sequences are represented; dots show individual graphs. The ordering—velocity residual highest, geometric diffusion second, both diffusion components of velocity and RCS lowest—is reproduced in essentially every scene (interquartile ranges below 0.0011), confirming that the near-uniform gate structure of Figure A1 is a property of the trained model rather than of one evaluation subset.

**Figure A3:**
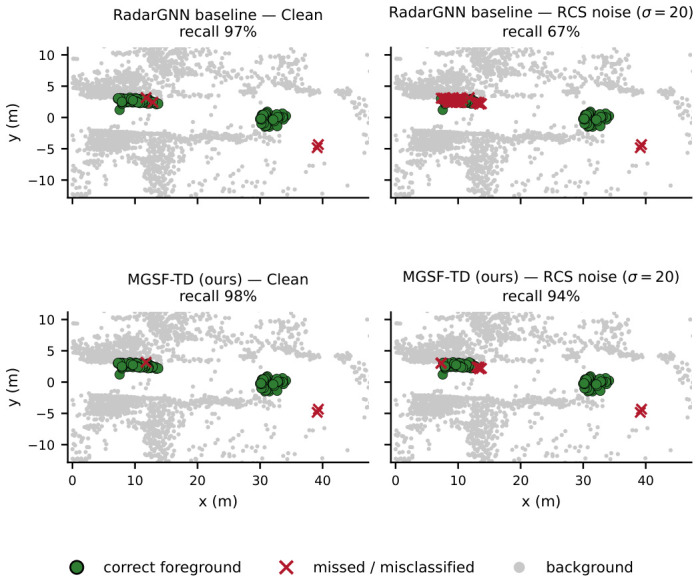
Point-level semantic predictions on a representative RadarScenes validation scene for RadarGNN (**top**) and MGSF-TD (**bottom**), under clean input (**left**) and RCS corruption at $\sigma_{\mathrm{rcs}}\;=\;20$ dBsm (**right**). Correct foreground predictions are green, missed or misclassified foreground points are red, and background points are gray. The scene is qualitative evidence only.

The spectral gate provides per-node context rather than the bulk of the discrimination. Class-wise mean gate weights (Appendix Figure A1), extracted from 200 validation graphs, stayed close to uniform at about 1/6 per component, with a slight preference for the residual velocity component among pedestrians and two-wheelers, which appear at velocity discontinuities. To verify that this pattern is not specific to one evaluation subset, we repeated the extraction over 500 graphs sampled with a uniform stride across the complete validation split, covering all recording sequences (Appendix Figure A2). The per-graph mean weights reproduce the same ordering in essentially every scene—velocity residual highest at about 0.183, velocity and RCS diffusion lowest at 0.154–0.155—with per-component interquartile ranges of at most 0.0011. The gate deviation from uniformity is therefore small but systematic and scene-stable. The learned gate was therefore not the dominant source of component differentiation. The component-specific projections produced a somewhat larger, although still modest, ablation effect, and uniform gate weights still underperform the learned gate.

Each MGSF block adds six component projections and a small gate MLP; across the five RadarGNN layers with d∈{224,224,128,64,32}, this amounts to about 0.87M backbone parameters over the 1.21M baseline (a 72% increase; 0.88M including the task-decoupled heads), with per-block cost $O(kNd+Nd^{2})$, which matches one message-passing layer. The task-decoupled heads of MGSF-TD then add only about 11k parameters beyond the full MGSF branch, below 1% of the total. The backbone addition dominates the inference cost: on a single GPU, MGSF-TD runs at 40.6 ms per graph (25 graphs/s) against 30.1 ms (33 graphs/s) for the baseline, a 35% latency overhead. This throughput exceeds the 2 Hz graph rate implied by the 500 ms RadarScenes accumulation window, but the overhead is material for embedded deployment. Stated as a trade-off, MGSF-TD spends about 0.88M additional parameters (+72%) and 10.5 ms per graph (+35%) to obtain +0.40 clean mAP, +1.04 clean FG-F1, and the degradation margins of Section 4.2. Whether this is practical depends on the deployment budget. At the 2 Hz graph rate of the accumulation window, MGSF-TD occupies well under a tenth of one GPU, so the overhead is immaterial in the evaluated configuration; on an embedded processor shared by several perception tasks, the same 35% competes directly with other workloads. Peak GPU inference memory is nearly unchanged—814 versus 811 MiB at batch size one over validation graphs, a 0.4% increase—because activation storage on the *k*-NN graph, not the added parameters, dominates the memory footprint. Reducing the number of GSOs, applying MGSF only to later layers, or pruning component projections are direct options when latency is tighter than in the present offline validation. We have not benchmarked automotive-grade embedded platforms; per-platform latency, memory, and energy profiling, including quantized and pruned variants, is a prerequisite for on-vehicle deployment and is left as engineering future work.

**Parameter-budget control.** To separate architectural structure from parameter count, we widen the plain RadarGNN backbone to convolution dimensions [300, 300, 168, 84, 42]. This WideCap control has 2.110M parameters, within 0.9% of MGSF-TD’s 2.091M, and is trained from scratch with seed 42 under the same data, input features, losses, and 30-epoch budget, using the best-validation checkpoint. Widening changes every hidden dimension, so the released RadarGNN checkpoint cannot be loaded directly; this control therefore matches parameter and training budgets but not initialization history. Under this training budget, WideCap does not reproduce the clean trade-off: it reaches 59.89 mAP and 73.94 FG-F1, below both the official baseline and MGSF-TD. It is also substantially weaker under position noise. However, it matches MGSF-TD at RCS σ=10 and exceeds it under severe RCS and Doppler corruption. Read plainly, Table 9 divides the regimes: MGSF-TD wins on clean data and mild corruption (clean mAP and FG-F1, RCS σ=3, position noise), WideCap wins under severe corruption (RCS σ=20, Doppler σ=2.0), and the two tie at RCS σ=10. These results rule out a capacity-only account of the mild-regime margins, and they equally rule out attributing severe-corruption tolerance to the physical operators; the control does not isolate initialization or optimization effects. We therefore interpret MGSF-TD as a balanced clean/degradation operating point rather than attributing every robustness gain uniquely to the physical operators.

### 4.8. Cross-Sensor Boundary Diagnostic

To probe where the operator-structure account holds beyond RadarScenes, we repeat the study on two further automotive radar datasets with different sensors, scene distributions, and class taxonomies: View-of-Delft (VoD) [50] (4D radar, official split of 5139 training and 1296 validation frames) and nuScenes radar [51] (five-radar setup, the first 4000 samples of v1.0-trainval, assigned deterministically by sample index to 3200 training and 800 validation graphs). Because both provide 3D boxes rather than point-level labels, we derive a point-level class for each return by testing whether it falls inside a ground-truth box, mapping car, pedestrian, and cyclist to three foreground classes and the rest to background. We build the same *k*-NN graphs (k=20) and node features as for RadarScenes (for VoD the 2D velocity is reconstructed from the compensated radial Doppler; nuScenes provides a 2D compensated velocity directly), train a RadarGNN baseline from scratch, fine-tune MGSF from it (single seed, best-validation checkpoints), and run the same corruption benchmark with foreground F1 averaged over the three present classes. The nuScenes split is sample-level and is used only as a diagnostic stress test, not as a formal scene-disjoint generalization benchmark. Table 10 summarizes the result against a dataset-native foreground-to-clutter RCS diagnostic

(6) $${\bar{c}}_{\mathrm{native}}=E_{\left(i,j\right)\in E_{\mathrm{fb}}}\;|r_{i}-r_{j}|-E_{\left(i,j\right)\in E_{\mathrm{ff}}}\;\left|r_{i}-r_{j}\right|,$$

the mean edge-level RCS difference on foreground-to-background edges $E_{\mathrm{fb}}$ minus that on same-class foreground edges $E_{\mathrm{ff}}$. This is a within-dataset diagnostic computed before the robustness evaluation. Because the datasets use their own RCS preprocessing and sensor conventions, the three values are not treated as calibrated physical dBsm quantities or as a quantitative cross-dataset threshold. Appendix Table A7 and Table A8 give the per-corruption VoD and nuScenes breakdowns.

MGSF produced clean FG-F1 offsets of +6.35 on VoD and +1.52 on nuScenes. The nuScenes mean was dominated by cars, while pedestrian and cyclist point-F1 remained near zero. We therefore treat these experiments as diagnostics rather than generalization benchmarks. The high-severity Doppler gap became negative on all three datasets. The RCS margin increased with severity on RadarScenes but decreased on VoD and nuScenes. Because the RCS diagnostic is dataset-native rather than calibrated across sensors, these results identify a boundary of the present operator account rather than a quantitative contrast threshold.

## 5. Discussion

**Degradation diagnostics and operating envelope.** The main outcome is not that one architecture dominates every corrupted setting. Rather, the multi-GSO construction gives an operator-level diagnostic for how radar measurements fail. MGSF-TD improved the joint semantic–localization trade-off without changing the RadarGNN candidate edge index or detection pipeline. The degradation experiments then showed that the relative margin depended on where noise entered the operator family. RCS noise altered both a node feature and the RCS-defined neighborhood, while the geometric and velocity operators retained independent structure. Positional noise perturbed the spatial coordinates and relative-position edge attributes shared by both models, but not the retained candidate edge index; accordingly, it produced no systematic margin. Doppler noise directly damaged an additional MGSF operator, causing the margin to disappear at high severity.

The controls qualify this operator-based interpretation. Geometry-only filtering accounted for part of the RCS gain and serves as a same-pipeline generic spectral smoothing control. The RCS GSO added a further increment, clearest at low-to-moderate RCS noise where edge weights still retain class contrast. Velocity augmentation recovered Doppler performance more strongly for RadarGNN than for MGSF, so Doppler robustness is not an MGSF capability. WideCap also exceeded MGSF-TD under severe RCS and Doppler corruption. Increased capacity, without any physical-operator structure, can therefore match or exceed the physics-guided module under extreme noise. It did not, however, reproduce the clean or positional operating point of MGSF-TD.

**The task-component conflict.** The ablations support a task-component conflict rather than a uniform benefit from additional operators. Doppler components improved foreground classification but weakened box regression when both heads shared one filtered representation. Separating the semantic and regression paths recovered the mAP loss while retaining the FG-F1 gain. Whether this principle transfers to other radar detectors requires evaluation under their own graph and head designs.

**Why multi-GSO is specific to radar.** LiDAR point clouds do not provide per-return Doppler velocity and RCS. The velocity and RCS GSOs therefore encode radar-specific sensing information that geometry alone cannot supply. MGSF leaves graph topology fixed and is compatible in principle with adaptive graph construction, including feature-space graphs such as DGCNN [34]. Their combination remains untested.

**Applicability beyond RadarGNN.** MGSF is defined at the level of a point-cloud graph rather than of one architecture: it requires only a per-point embedding, a candidate neighborhood structure, and per-point Doppler and RCS measurements. In principle it can be inserted into other graph detectors as an additional residual stage, and its attribute-defined operators could enter transformer- or pillar-based radar detectors as attention biases or per-pillar reweighting. We evaluate it on RadarGNN because the public official checkpoint permits a controlled fixed-pipeline comparison that other radar graph detectors do not currently offer; transferring the module to further backbones is left as future work rather than claimed. What makes such retrofitting safe is the identity-initialized residual design: with β=0 the block is an exact identity at insertion, so any pre-trained detector is preserved until fine-tuning moves it. We emphasize this under-reported pattern as a reusable design element in its own right, independent of the radar-specific operators.

**Why behavior differs across datasets.** The cross-sensor differences in Table 10 have identifiable sources. First, the dataset-native RCS contrast between foreground-to-background and same-class edges decreases from RadarScenes (2.65) to View-of-Delft (1.89) to nuScenes (1.25), and the severity-amplified RCS margin appears only at the highest contrast: when class-discriminative RCS structure is weak, the RCS operator has little signal to preserve under corruption. Second, the cross-sensor labels are box-derived rather than point-annotated, and the nuScenes foreground mean is dominated by cars, so absolute margins are not comparable across datasets. Third, the cross-sensor baselines are from-scratch, single-seed models at operating points different from the official RadarScenes checkpoint. The deployment implication is direct: operator-level benefits should be validated per sensor configuration before use, and the native RCS-contrast diagnostic—computable from box-labeled data before any training—is a candidate low-cost predictor of whether the RCS operator will help on a new sensor.

**Figure A4:**
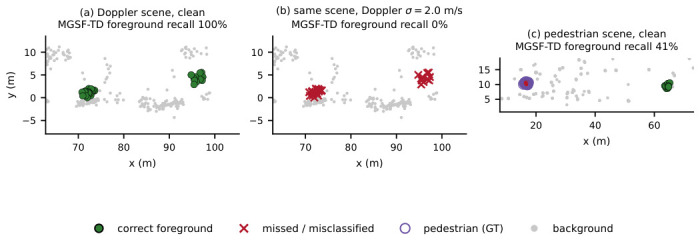
Qualitative failure cases of MGSF-TD (seed 42), selected by fixed rules over the first 200 validation scenes rather than by hand. (**a**,**b**) The scene with the largest foreground-recall drop under Doppler noise among scenes with clean recall above 80%: recall falls from 100% to 0% at $\sigma_{\mathrm{vel}}\;=\;2.0$ m/s, illustrating the declared Doppler failure boundary. (**c**) The scene with the most pedestrian points among scenes where at least half of the pedestrian points are misclassified when clean (purple circles mark ground-truth pedestrian points): the pedestrian cluster is absorbed into clutter while the vehicle cluster is detected, illustrating the pedestrian weakness discussed in Section 5.

**Limitations and failure modes.** Several limitations bound these conclusions. Pedestrian AP remained below the RadarGNN baseline, consistent with the task-component conflict rather than with a uniform gain from added operators. Pedestrians are often spatially close to static clutter, and the fixed spatial *k*-NN candidate graph can include off-class neighbors before any velocity or RCS reweighting is applied. Task decoupling reduces the semantic–regression conflict but does not change that candidate topology. A class- or motion-aware topology may therefore be needed instead of reweighting a fixed spatial graph. The diagnosis suggests a concrete mitigation: augment the candidate graph with a small number of velocity-gated edges, connecting only returns whose compensated speeds both exceed a clutter threshold, so that pedestrians acquire same-class neighbors before any reweighting, while box regression keeps the geometric graph. Combining this with the existing task decoupling targets the pedestrian AP deficit without altering the operators studied here; we outline it as the next design iteration rather than evaluating it in this revision. Strong Doppler corruption also remained a failure mode, and its augmentation-based recovery reduced clean and positional accuracy. Appendix Figure A4 illustrates both failure modes on rule-selected validation scenes.

These failure modes carry direct safety implications for autonomous driving. The pedestrian AP deficit and the Doppler corruption boundary together mean that MGSF-TD should not serve as a sole detection source for vulnerable road users: a corrupted Doppler channel degrades the velocity operator precisely for the slow-moving classes it is meant to separate from clutter. We therefore position the module as one redundant component inside a multi-sensor perception stack with independent camera or LiDAR paths and with online degradation monitoring, not as a standalone safety function. Overstating robustness is itself a societal risk; the capacity control shows that part of the extreme-severity margin can be matched by generic capacity, which is why the boundary conditions are reported explicitly rather than folded into a single headline gain. The evaluated corruptions also exclude scene-coupled effects such as multipath ghost targets, mutual interference, and weather-dependent attenuation, so the present results bound behavior under attribute-level noise only. For the same reason, evaluation on naturally degraded recordings remains open: adverse-weather 4D-radar datasets such as K-Radar [22] provide rain, fog, and snow sequences, and testing whether the RCS-operator margin persists under natural degradation, rather than injected noise, is the most direct next step toward practical robustness evidence.

The cross-sensor experiments provide diagnostic evidence only. Their point labels were derived from boxes, and the nuScenes foreground mean was dominated by cars. Moreover, severity-dependent RCS gains did not persist across the two box-derived cross-sensor diagnostics. More sensors and backbones are required to test whether a calibrated RCS-contrast relationship exists. The diagnostic nuScenes split is sample-level and may retain scene-level or temporal correlations between its training and validation subsets; its absolute gains should therefore not be interpreted as scene-disjoint generalization performance. Finally, clean detection, ablations, capacity control, and full-validation RCS mAP use fixed checkpoints. Their differences are descriptive rather than estimates of training-seed significance. The three-seed robustness analysis varies MGSF-TD but retains one official RadarGNN checkpoint, and the graph-level bootstrap measures evaluation-set uncertainty rather than training variance; the bootstrap does not explicitly model correlations among graphs originating from the same recording sequence.

## 6. Conclusions

MGSF introduces physics-defined geometric, Doppler, and RCS operators into automotive radar point-cloud detection. Its task-decoupled variant improves the clean semantic and localization trade-off under the RadarGNN pipeline. More importantly, the operator decomposition provides a diagnostic view of sensor degradation: RCS corruption benefits from both generic diffusion smoothing and an RCS-defined neighborhood contribution, positional noise is largely common-mode, and strong Doppler noise marks a failure boundary of the velocity operator.

The evidence supports MGSF-TD as a balanced and physically interpretable operating point. The parameter-budget control shows that additional capacity can outperform MGSF-TD under extreme corruption, although it does not recover the clean or positional operating point. Pedestrian localization and cross-sensor RCS behavior remain the principal unresolved limitations.

Future work concerns deployment. Embedding MGSF-TD in a vehicle requires latency- and memory-bounded implementations on automotive processors—the measured 35% latency overhead is affordable at the 2 Hz graph rate of this study but is a real cost in a shared real-time perception stack—integration with camera and LiDAR fusion [30] under vehicle-level sensor-suite selection and placement constraints [52], and validation against scene-coupled degradations such as ghost targets and interference. These steps interact with the broader, well-documented systems-level challenges of fielding autonomous-vehicle perception—sensor limitations, robustness under operational conditions, and integration constraints [53]. Within that larger picture, this paper contributes a physically interpretable, degradation-aware filtering module intended as one building block of deployable radar perception in intelligent transportation systems.

## Figures and Tables

**Table 1:** Positioning of MGSF against representative graph spectral filtering, multi-graph, and graph detection methods.

Method	Graph Construction	Filtering Strategy	Robustness Objective
ChebNet [8], GPR-GNN [40], BernNet [9]	Single given graph	One polynomial spectral filter shared by all nodes	None (clean accuracy)
DGCNN [34]	Feature-space *k*-NN, rebuilt per layer	Spatial message passing on the dynamic graph	None
MAGNN [43]	Heterogeneous metapath multi-graph	Per-metapath aggregation with attention	None (heterogeneous semantics)
Point-GNN [5], RadarGNN [3]	Single spatial neighborhood graph	Message passing; one shared representation for both heads	None (clean detection)
**MGSF-TD (ours)**	Fixed spatial *k*-NN plus three physics-defined GSOs (geometry, Doppler, RCS)	Per-GSO diffusion–residual decomposition, per-node gated fusion, task-decoupled heads	Explicit: operator-level degradation diagnostics and margins

**Table 2:** Degradation models and their physical interpretation. The idealized modes isolate individual attributes, the range-dependent polar jitter provides a sensor-structured localization model, and point dropout serves as a generic sparsity stress test. Severity ranges span mild-to-severe degradation in the native unit of each attribute.

Corruption	Model	Physical Interpretation	Severity Rationale
RCS noise	Additive Gaussian (dBsm)	Log-domain reflectivity fluctuation from aspect angle, target material, multipath, and weather	3–20 dBsm, mild-to-severe reflectivity instability
Position (idealized)	Isotropic xy Gaussian (m)	Localization error from finite range/azimuth resolution	0.1–1.0 m, sub-resolution to multi-cell
Position (realistic)	Range-dependent polar jitter	Range and azimuth uncertainty; angular noise scaled by graph median range	σ=0.2–1.0 m range noise with matched cross-range scale
Doppler	Additive velocity noise (m/s)	Ego-motion-compensation residual, radial-to-2D reconstruction, multipath, aliasing	0.5–2.0 m/s; large values are diagnostic stress tests
Point dropout	Random node removal	Generic missed-return sparsification; not a calibrated RCS/SNR detection model	10–40% of points removed

**Table 3:** Main results on RadarScenes validation with exact rotated NMS. RadarGNN model_02 is re-evaluated as the fair baseline. All MGSF variants use seed 42 and calibrated $\sigma_{\mathrm{vel}}\;=\;1.0$ m/s. These clean results are seed-42 values; the MGSF-TD clean FG-F1 mean over three seeds is 75.02±0.15. Boldface identifies the selected proposed configuration and its corresponding values.

Method	mAP	ΔmAP	FG-F1	ΔF1
RadarGNN model_02 [3]	60.19	—	74.06	—
MGSF Geo	60.69	+0.50	74.58	+0.52
MGSF Full (3-GSO)	59.86	−0.33	75.09	+1.03
**MGSF-TD (ours)**	**60.59**	+0.40	**75.10**	+1.04

**Table 4:** Robustness under single-attribute sensor degradation (FG-F1, %, no retraining). MGSF-TD is the mean ± standard deviation over three training seeds (42, 7, 123); the baseline is the official RadarGNN checkpoint (its clean value matches Table 3). The gap is $\Delta_{\mathrm{rob}}={\mathrm{FG}\text{-}F1}_{MGSF}^{\mathrm{mean}}-{\mathrm{FG}\text{-}F1}_{\mathrm{base}}$. The RCS margin grows with severity while the positional gap stays within the MGSF-TD seed spread. Gaps are computed from unrounded values and then rounded. The graph-level bootstrap reported in the text is a separate fixed-checkpoint analysis for the seed-42 model pair, not a confidence interval for the three-seed mean gap. Velocity-augmentation controls are reported in Appendix A, Table A1. Boldface marks the largest positive robustness margin in the table.

Corruption	σ	Base (Official Ckpt)	MGSF-TD (Seeds 42/7/123)	$\Delta_{\mathrm{rob}}$
Clean	—	74.06	75.02±0.15	+0.96
RCS (dBsm)	3	73.57	74.72±0.11	+1.15
	10	68.43	69.91±0.25	+1.48
	20	59.09	61.47±0.46	+2.38
Position (m)	0.1	58.15	58.46±0.67	+0.30
	0.2	57.22	57.54±0.62	+0.32
	0.5	52.61	52.52±0.57	−0.09
	1.0	44.54	45.15±0.96	+0.62
Doppler (m/s)	0.5	44.11	45.94±0.77	+1.83
	1.0	31.40	31.64±0.85	+0.24
	2.0	23.77	23.13±0.61	−0.63

**Table 5:** RCS-degradation decomposition by GSO subset (FG-F1 gap vs. baseline, $\Delta_{\mathrm{rob}}$, %, seed 42). The geo-only model already grows; the velocity GSO is neutral-to-negative under RCS noise; the RCS GSO adds an increment beyond generic diffusion smoothing, most cleanly at low-to-moderate severity. Boldface identifies the derived increment contributed by the RCS GSO.

GSO Subset	$\sigma_{\mathrm{rcs}}\;=\;3$	10	20
Geo only	+0.72	+1.11	+1.90
Geo + Vel	+0.69	+0.49	+1.27
Geo + Vel + RCS (full)	+1.25	+1.74	+2.76
**RCS-GSO increment**	+0.56	+1.25	+1.49

**Table 6:** Detection mAP under RCS corruption (%, complete 5003-graph RadarScenes validation split, exact rotated NMS, fixed checkpoints without retraining: official RadarGNN and seed-42 MGSF-TD). Boldface marks the higher mAP within each paired comparison.

$\sigma_{\mathrm{rcs}}$ (dBsm)	Base mAP	MGSF-TD mAP	ΔmAP
Clean (0)	60.19	**60.59**	+0.40
3	59.81	**60.05**	+0.24
10	54.83	**55.91**	+1.07
20	46.09	**47.91**	+1.82

**Table 7:** Sensor-structured and sparsity degradation (FG-F1, %, RadarScenes validation, models evaluated without retraining; baseline = official checkpoint, MGSF-TD = seed 42). Range-dependent jitter is common-mode (small gap, as for idealized position); point dropout leaves MGSF-TD a stable positive margin.

Corruption	Severity	Base	MGSF-TD	$\Delta_{\mathrm{rob}}$
Range-dep. jitter (m)	0.2	57.93	58.60	+0.67
	0.5	54.93	55.36	+0.43
	1.0	50.96	51.20	+0.24
Point dropout (frac.)	0.1	73.53	74.58	+1.05
	0.2	72.79	74.00	+1.21
	0.4	69.93	71.13	+1.20

**Table 8:** Ablation study (seed 42). Δ values are relative to the RadarGNN baseline (60.19/74.06). $\sigma_{\mathrm{vel}}^{*}$: calibrated $\sigma_{\mathrm{vel}}\;=\;1.0$ m/s. Boldface marks the best value in the corresponding metric column or the selected task-decoupled configuration.

Configuration	mAP	ΔmAP	FG-F1	ΔF1
RadarGNN baseline	60.19	—	74.06	—
MGSF Geo only	60.69	+0.50	74.58	+0.52
+ Vel GSO ($\sigma_{\mathrm{vel}}^{*}$)	60.08	−0.11	74.68	+0.62
+ RCS GSO ($\sigma_{\mathrm{vel}}^{*}$)	59.86	−0.33	**75.09**	+1.03
w/o gate (uniform)	59.74	−0.45	75.00	+0.94
w/o component-specific proj.	59.66	−0.53	75.05	+0.99
MGSF-TD ($\sigma_{\mathrm{vel}}^{*}$)	**60.59**	+0.40	75.10	+1.04

**Table 9:** Parameter-budget control (%, fixed checkpoints: official RadarGNN, seed-42 MGSF-TD, and seed-42 WideCap). WideCap is a widened plain RadarGNN, not an MGSF variant, with 2.110M parameters versus 2.091M for MGSF-TD. FG-F1 is reported except where mAP is specified. Boldface marks the best value in each condition.

Condition	RadarGNN	MGSF-TD	WideCap
Clean mAP	60.19	**60.59**	59.89
Clean FG-F1	74.06	**75.10**	73.94
RCS σ=3	73.57	**74.82**	73.67
RCS σ=10	68.43	70.17	**70.18**
RCS σ=20	59.09	61.85	**64.28**
Position σ=1.0	44.54	**45.94**	40.49
Doppler σ=2.0	23.77	22.72	**36.56**

**Table 10:** Cross-sensor boundary diagnostic summary. For each radar dataset, we report a dataset-native RCS diagnostic (mean edge-level foreground–background |ΔRCS| minus same-foreground |ΔRCS|), the clean foreground-F1 gain over its corresponding baseline, the low-to-high-severity RCS robustness gap, its trend, and the Doppler velocity gap at the strongest tested severity. RadarScenes uses the official RadarGNN checkpoint and the three-seed MGSF-TD mean, whereas the View-of-Delft and nuScenes diagnostics use from-scratch, single-seed baseline–MGSF pairs. These results are hypothesis-generating diagnostics rather than generalization benchmarks.

Dataset	Native RCS Diag.	Clean Δ	RCS Gap (lo→hi)	Trend	Vel Gap
RadarScenes	2.65	+0.96	+1.15→+2.38	grows	−0.63
View-of-Delft	1.89	+6.35	+5.91→+1.71	shrinks	−3.15
nuScenes	1.25	+1.52	+1.45→+0.39	shrinks	−0.19

## Data Availability

This study uses three publicly available automotive radar datasets: RadarScenes [2] (https://radar-scenes.com, accessed on 24 July 2026), View-of-Delft [50] (https://github.com/tudelft-iv/view-of-delft-dataset, accessed on 24 July 2026), and nuScenes [51] (https://www.nuscenes.org, accessed on 24 July 2026). Additional numerical results are reported in Appendix A. The complete source code, experiment configurations, corruption generation and robustness evaluation scripts, training-seed declarations, and all trained checkpoints used for the reported RadarScenes, WideCap, View-of-Delft, and nuScenes experiments are openly archived on Zenodo at https://doi.org/10.5281/zenodo.21333919.

## Abbreviations

The following abbreviations are used in this manuscript:

MMW	Millimeter-wave
RCS	Radar cross-section
GNN	Graph neural network
GSP	Graph signal processing
GSO	Graph shift operator
MGSF	Multi-GSO spectral filtering
MGSF-TD	Task-decoupled MGSF
BEV	Bird’s-eye view
mAP	Mean average precision
FG-F1	Mean foreground F1
NMS	Non-maximum suppression

## Appendix A. Supporting Analyses

This appendix reports supporting diagnostics and detailed breakdowns referenced in the main text. They are included in the article PDF to keep the evidence self-contained while preserving the main Section 4’s focus on the primary detection, robustness, mechanism, and capacity-control findings.

**Table A1:** Velocity-augmentation control (FG-F1, %, single best-validation checkpoint). MGSF+Aug and RGNN+Aug use the same velocity augmentation. In the continued part, boldface marks the best value in each Doppler-corruption row.

Corruption	σ	Base	MGSF+Aug	RGNN+Aug
Clean	—	74.06	74.04	73.06
RCS (dBsm)	3	73.57	73.55	72.49
	10	68.43	67.44	67.65
	20	59.09	58.11	58.10
Position (m)	0.1	58.15	57.54	56.48
	0.5	52.61	51.77	47.11
	1.0	44.54	44.76	37.94
Doppler (m/s)	0.5	44.11	65.88	**69.88**
	1.0	31.40	59.61	**65.21**
	2.0	23.77	49.80	**57.62**

**Table A2:** RCS-GSO edge weights, clean versus RCS σ=20 dBsm corruption (ff: intra-class foreground; fb: foreground–background; bb: background–background). Values are raw kernel weights $w_{ji}^{\mathrm{rcs}}$ averaged over all edges of each type, computed before degree normalization.

Edge Type	Clean Weight	Corrupted Weight
ff	0.621	0.249
fb	0.517	0.242
bb	0.645	0.248

**Table A3:** Per-class AP at RCS σ=20 dBsm (%, complete 5003-graph RadarScenes validation split). Boldface marks the higher AP within each paired comparison.

Class	Base	MGSF-TD	Δ
Car	58.33	**60.99**	+2.66
Pedestrian	18.32	**18.52**	+0.20
Pedestrian group	35.69	**37.45**	+1.76
Two-wheeler	51.39	**57.59**	+6.20
Large vehicle	**66.73**	64.98	−1.75
mAP	46.09	**47.91**	+1.82

**Table A4:** Clean per-class AP (%, seed 42). Boldface marks the highest mAP among the controlled variants and the selected MGSF-TD result.

Class	Baseline	Geo-Only	Full	MGSF-TD
Car	71.97	74.70	74.08	73.65
Pedestrian	33.96	34.56	31.58	31.93
Pedestrian group	58.28	58.17	58.43	58.34
Two-wheeler	66.58	65.84	65.27	68.42
Large vehicle	70.15	70.16	69.94	70.59
mAP	60.19	**60.69**	59.86	**60.59**

**Table A5:** Bandwidth sensitivity of the full three-GSO model (clean detection mAP, %, seed 42, RadarScenes validation). Each non-bold row retrains the model with one bandwidth changed relative to the selected configuration ($\sigma_{\mathrm{vel}}\;=\;1.0$ m/s, $\sigma_{\mathrm{rcs}}\;=\;10$ dBsm, bold row); $\sigma_{\mathrm{geo}}\;=\;4.0$ m is fixed throughout. mAP varies by at most 0.39 points across the grid.

$\sigma_{\mathrm{vel}}$ (m/s)	$\sigma_{\mathrm{rcs}}$ (dBsm)	mAP
0.5	10	59.75
**1.0**	**10**	**59.86**
2.0	10	59.80
3.0	10	59.60
1.0	3	59.47
1.0	5	59.81

**Table A6:** From-scratch baseline seed control (FG-F1, %, except the first row). Two plain RadarGNN baselines are retrained from scratch (seeds 7 and 123) under the same data, features, losses, and 30-epoch best-validation schedule as the WideCap control and evaluated on the identical corruption realizations. Seed 123 (best validation at epoch 20) closely reproduces the official checkpoint’s degradation profile at lower clean accuracy; seed 7 stops at an earlier validation optimum (epoch 14) and trades about five clean FG-F1 points for flatter degradation, most visibly under Doppler noise. MGSF-TD is the three-seed mean of Table 4 (clean mAP: seed-42 value) and is fine-tuned from the official checkpoint, so the official column remains its paired reference. Boldface marks the highest value in each reported condition.

Condition	σ	Official	Seed 7	Seed 123	MGSF-TD
Clean mAP	—	60.19	56.79	57.36	**60.59**
Clean FG-F1	—	74.06	68.88	72.49	75.02±0.15
RCS (dBsm)	3	73.57	68.73	72.16	74.72±0.11
	10	68.43	65.76	68.52	69.91±0.25
	20	59.09	60.42	59.16	61.47±0.46
Position (m)	0.1	58.15	57.15	60.37	58.46±0.67
	0.2	57.22	54.52	58.00	57.54±0.62
	0.5	52.61	47.12	51.36	52.52±0.57
	1.0	44.54	40.95	45.36	45.15±0.96
Doppler (m/s)	0.5	44.11	57.02	51.30	45.94±0.77
	1.0	31.40	48.29	34.10	31.64±0.85
	2.0	23.77	37.96	23.86	23.13±0.61

**Cross-sensor diagnostic protocol.** View-of-Delft uses its official 5139-frame training split and 1296-frame validation split. nuScenes uses the first 4000 samples of v1.0-trainval; every fifth sample is assigned to validation, yielding 3200 training and 800 validation graphs. This nuScenes split is deterministic but sample-level, so it is not used as a formal scene-disjoint benchmark. These diagnostics use single-seed baseline–MGSF pairs (seed 42), batch size 5, Adam training, and best-validation checkpoints. A radar point is assigned a foreground label if it lies inside a foreground 3D box; if multiple boxes contain the same point, the first matched foreground box in the dataset order is used. VoD graphs are built from the provided 5-frame radar representation, while nuScenes graphs merge the five radar channels of one sample into the reference ego frame. FG-F1 is the unweighted mean over the foreground classes present in the diagnostic label set; absent classes are not included in the mean. RCS values are used in the dataset units available after preprocessing, so the cross-sensor RCS diagnostic is treated as within-dataset evidence rather than a calibrated cross-dataset physical threshold.

**Table A7:** Per-corruption View-of-Delft results (FG-F1, %, single seed, best-validation checkpoints). RCS perturbation magnitudes are expressed in this dataset’s native preprocessed units and are not physically matched across sensors.

Corruption	σ	Base	MGSF	Δ
Clean	—	39.14	45.49	+6.35
RCS noise (native units)	3	38.86	44.77	+5.91
	10	34.62	39.30	+4.68
	20	26.54	28.25	+1.71
Position (m)	0.1	38.47	44.74	+6.27
	0.5	32.67	36.95	+4.28
Doppler (m/s)	0.5	27.94	31.03	+3.09
	2.0	22.14	18.99	−3.15

**Table A8:** Per-corruption nuScenes results (FG-F1, %, single seed, best-validation checkpoints). The foreground mean is dominated by the car class. RCS perturbation magnitudes are expressed in this dataset’s native preprocessed units and are not physically matched across sensors.

Corruption	σ	Base	MGSF	Δ
Clean	—	7.61	9.13	+1.52
RCS noise (native units)	3	7.60	9.05	+1.45
	10	7.29	8.34	+1.05
	20	6.77	7.16	+0.39
Position (m)	0.1	7.59	9.13	+1.54
	0.5	7.56	9.05	+1.49
Doppler (m/s)	0.5	4.60	4.39	−0.21
	2.0	3.23	3.04	−0.19
